# Triple-Model Immunoassays with the Self-Assemblies of Three-in-One Small Molecules as Signaling Labels

**DOI:** 10.3390/bios15110710

**Published:** 2025-10-24

**Authors:** Zhaojiang Yu, Wenqi Yuan, Mingyi Qiao, Lin Liu

**Affiliations:** College of Chemistry and Chemical Engineering, Anyang Normal University, Anyang 455000, China; yzj86jiang@aynu.edu.cn (Z.Y.); 19638752312@163.com (W.Y.); qiaomingyi2003@163.com (M.Q.)

**Keywords:** self-assembly, biorecognition, multiple-mode immunoassays, pyrroloquinoline quinone, signal amplification

## Abstract

Multiple-mode immunoassays have the advantages of self-correction, self-validation, and high accuracy and reliability. In this work, we developed a strategy for the design of triple-mode immunoassays with the self-assemblies of three-in-one small molecules as signal reporters. Pyrroloquinoline quinone (PQQ), with a well-defined redox peak and excellent spectroscopic and fluorescent signals, was chosen as the signaling molecule. PQQ was coordinated with Cu^2+^ to form metal–organic nanoparticle as the signal label. Hexahistidine (His_6_)-tagged recognition element (recombinant streptavidin) was attached to the Cu-PQQ surface through metal coordination interaction between the His_6_ tag and the unsaturated metal site. The captured Cu-PQQ nanoparticle released a large number of PQQ molecules under an acidic condition, which could be simultaneously monitoring by electrochemical, UV-vis, and fluorescent techniques, thereby allowing for the development of triple-model immunoassays. The three methods were used to determine the concentration of carcinoembryonic antigen (CEA) with the detection limits of 0.01, 0.1, and 0.1 ng/mL, respectively. This strategy opens up a universal route for the preparation of multiple-model signal labels and the oriented immobilization of bioreceptors for molecular recognition.

## 1. Introduction

Immunoassays, a category of popular analytical methods that employ antibodies as bioreceptors and/or recognition elements, have found wide applications in the fields of disease diagnosis, pollutant analysis, and food safety [1,2,3]. They are typically classified as electrochemical, fluorescent, colorimetric, chemiluminescent, and surface-enhanced Raman scattering (SERS) immunoassays, among other types [1,4]. To date, immunoassays are mainly designed and used in different fields with a single-signal detection mode, which offers the advantages of ease of operation and rapid detection. However, the results of single-signal immunoassays are sensitive to various factors, such as different experimental conditions, non-standard analysis procedures, and unskilled operators [5]. On the contrary, the multiple-signal detection mode, combining the advantages of different single-signal technologies, can expand the linear range, perform self-verification, and improve detection diversity and precision [6,7,8].

Recently, the development of multiple-signal detection modes with self-correction and self-validation capabilities has attracted widespread attention from researchers. Multiple-readout immunoassays can avoid fluctuations in analysis data, enhance mutual verification, and ultimately improve the detection accuracy and reliability [9,10,11,12,13]. The methods typically require two or more different techniques to collect the signals, involving different signal reporters or multifunctional nanomaterials. Generally, some nanomaterials and enzyme- or nanozyme-catalyzed products can serve as redox signal molecules, chromogenic substrates, surface-enhanced Raman spectroscopy (SERS) reporters, and fluorophores or quenchers for a multiple-signal readout [5]. For example, gold- or silver-based nanoparticles were used as both plasmonic chromogenic substrates and fluorescence quenchers of dual-signal biosensors. The oxidized format of 3,3′,5,5′-tetramethylbenzidine (TMB) has served as both indicator and quencher for the fabrication of colorimetric–fluorescence dual-model immunoassays. In addition, some dual-functional organic molecules, such as fluorescein and pH indicators, have been loaded into nanocarriers to serve as the signal reporters of dual-signal immunoassays [5,14]. In contrast to nanomaterials and enzymes or nanozymes, the self-assemblies of small organic molecules could serve as signaling reporters with intrinsic advantages (e.g., simple preparation, high scalability, good stability, and signal multiplicity) [14]. After the immunoreaction, plenty of signaling molecules could be eluted for signal-amplified output. For this purpose, self-carried nanomaterials have been prepared via the assembly of hydrophobic organic molecules and used as the signal labels for immunoassays, including TMB, pH indicators, tetra(4-carboxyphenyl)porphyrin, and phenylalanine derivatives [15,16,17,18]. The methods are sensitive and feasible, but most of the small molecules generate single signals. To the best of our knowledge, there are few reports on using self-assemblies of small molecules with triple-signal characteristics as signaling reporters for the development of multiple-readout immunoassays.

Pyrroloquinoline quinone (PQQ) is a redox-active cofactor of bacterial dehydrogenases. It exists in a variety of dietary sources and shows a well-defined redox peak and excellent UV-vis and fluorescent signals. The triple-signal characteristic theoretically endows PQQ with the ability to act as a signaling molecule for multiple-readout immunoassays. However, the sensitivity of such immunoassays would be much lower than that of the standard enzyme-linked immunosorbent assays if PQQ were directly attached as a label to the antibody as the signal reporter. The metal-coordinated self-assembly of organic molecules through Lewis acid/base interactions has developed into an elegant strategy for manufacturing precision therapeutic nanosystems [19,20]. The metal coordination bonds exhibit stable and dynamic behavior in complicated environments, as their strength falls within the range of weak non-covalent interactions and strong covalent bonds. For example, natural products with functional groups (hydroxyl, amino, carbonyl, etc.) containing oxygen and nitrogen atoms have usually been used as the coordination sites of metal ions (Fe^2+^/^3+^, Cu^+^/^2+^, Zn^2+^, Ni^2+^, and others) to form stable metal–organic hybrids for the diagnosis and treatment of tumors [21]. Metal–phenolic networks (MPNs) formed via coordination interactions between metal ions and phenolic ligands show antioxidant and antibacterial properties and have received widespread attention in view of their facile synthesis processes, excellent biocompatibility, and excellent antimicrobial activities provided by both metal ions and polyphenols [22,23,24]. PQQ contains three carboxyl groups that can serve as the building sites for coordination with metal ions to form metal–organic hybrids. In this work, we first investigated the assembly behavior of PQQ into nanoarchitectures in the presence of different metal ions (e.g., Cu^2+^, Zn^2+^, Ni^2+^, Fe^3+^, and Al^3+^) and found that the Cu-PQQ hybrid exhibits a uniform nanosphere structure. The Cu-PQQ nanoparticle could be disassembled by an acidic solution, thereby releasing a large number of PQQ molecules. Inspired by these results, Cu-PQQ nanoparticle was used as the signal label of triple-readout immunoassay in view of the excellent electrochemical, spectroscopic, and fluorescent properties of PQQ. The Cu-PQQ nanoparticle attached to the sensor surface through sandwich immunoreaction could release a large number of PQQ molecules under acidic conditions. The released PQQ can be simultaneously determined by electrochemical, UV-vis, and fluorescent techniques, thus allowing for the development of triple-readout immunoassays of the target. To prove the feasibility of this strategy, carcinoembryonic antigen (CEA) was determined as an example analyte with high sensitivity, simplicity, and accuracy. This work can provide inspiration for researchers to develop multiple-mode detection platforms and prepare fascinating sensing materials, including the preparation of multiple-signal labels via the self-assembly of small signaling molecules and the oriented immobilization of bioreceptors for molecular recognition and signal readout.

## 2. Materials and Methods

### 2.1. Reagents and Instruments

The experimental reagents and instruments are shown in Supplementary Materials.

### 2.2. Preparation of Metal-PQQ Hybrids

First, 200 mg of PVP was dissolved in a mixed solvent consisting of 4 mL of DMF and 4 mL of ethanol to obtain solution A. Then, 23.2 mg of copper nitrate and 5.4 mg of PQQ were dissolved in 4 mL of DMF to obtain solution B. Solutions A and B were mixed under mild sonication for 20 min, and then the mixture was transferred into a polytetrafluoroethylene reaction kettle, where it was heated at 100 °C for 8 h. After cooling to room temperature, the Cu-PQQ hybrids were obtained by centrifuging, washing, and drying them in a vacuum in turn. Zn-PQQ, Ni-PQQ, Fe-PQQ, and Al-PQQ were synthesized using a procedure similar to that of Cu-PQQ synthesis, except copper nitrate was replaced by zinc nitrate (36.7 mg), nickel nitrate (36 mg), iron nitrate (49.8 mg), or aluminum nitrate (46.28 mg).

### 2.3. Modification of Cu-PQQ with Recombinant Streptavidin (rSA)

Recombinant protein rSA with a hexahistidine (His_6_) tag was used as the linker of biotinylated detection antibody (Ab_2_–biotin) and Cu-PQQ through the classical avidin–biotin coupling system. It was attached to the Cu-PQQ surface through the coordination interaction between the His_6_ tag in rSA and the unsaturated metal site on Cu-PQQ [25]. Briefly, 0.1 mg of Cu-PQQ powder was dispersed in 1 mL of phosphate buffer (10 mM, pH 7.4). After sonication for 10 min, 1 mL of 1 μg/mL rSA solution was mixed with the Cu-PQQ suspension. After shaking for 60 min, the rSA-modified Cu-PQQ (rSA@Cu-PQQ) was centrifugally washed three times with phosphate buffer. The precipitates of rSA@Cu-PQQ were dispersed in 10 mL of phosphate-buffer solution for use.

### 2.4. Procedures for CEA Detection

The capture antibody (Ab_1_)-covered 96-well microplates were incubated with 100 μL of different concentrations of CEA for 30 min at room temperature. After being thoroughly rinsed with phosphate-buffer solution, the CEA-treated microplates were incubated with 100 μL of Ab_2_–biotin (100 ng/mL) for an additional 30 min. After washing the microplates with phosphate-buffer solution three times, 100 μL of the prepared rSA@Cu-PQQ was added. After incubation at room temperature for another 30 min, the microplates were washed three times. Finally, 100 μL of 50 mM acetic acid buffer solution containing 5% DMF (*v*/*v*) was added to the microplates. The solutions were collected for electrochemical, UV-vis, and fluorescent measurements. The excitation wavelength for the fluorescence assays was 340 nm. The detection limit was obtained by determining the minimum target concentration that can be apparently distinguished from that of the background control.

## 3. Results and Discussion

### 3.1. Characterization of Metal-PQQ Hybrids

**Figure 1:**
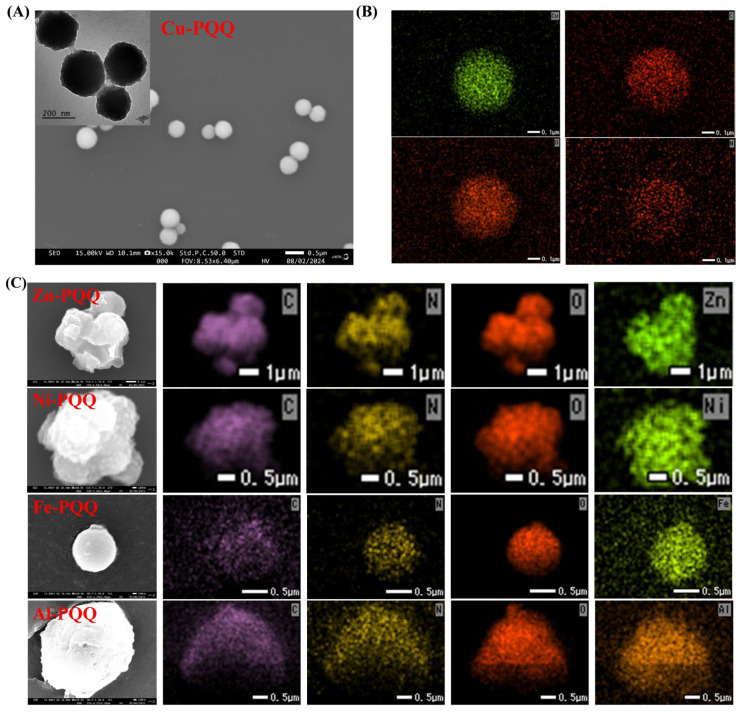
SEM image (**A**) and elemental mapping (**B**) of Cu-PQQ. The inset in panel A shows the transmission electron microscopy (TEM) image of Cu-PQQ. (**C**) Elemental mapping images of Zn-PQQ, Ni-PQQ, Fe-PQQ, and Al-PQQ.

The adjustable functionalities and morphologies of nanostructures should be considered when they are used as the signal labels of biosensors. In this study, metal–PQQ hybrids were prepared via coordination interactions between metal ions and PQQ molecules. We found that Cu^2+^ could be readily coordinated with PQQ to form supramolecular nanostructures (Figure 1A), while the other metal ions, Zn^2+^, Ni^2+^, Fe^3+^, and Al^3+^, did not form uniform nanostructures. Elemental mapping confirmed the presence of metal, carbon, nitrogen, and oxygen in the metal–PQQ hybrids (Figure 1B,C). In addition, the size of Cu-PQQ was found to be about 500 nm, as measured by dynamic light scattering. This size is larger than that observed using a scanning electron microscope (SEM), which is understandable since dynamic light scattering is used to measure the hydrodynamic size but not the solid diameter of nanoparticles. In this work, Cu-PQQ nanoparticles were used as signal labels for the development of triple-readout immunoassays in view of their good dispersibility and homogeneous size.

**Figure 2:**
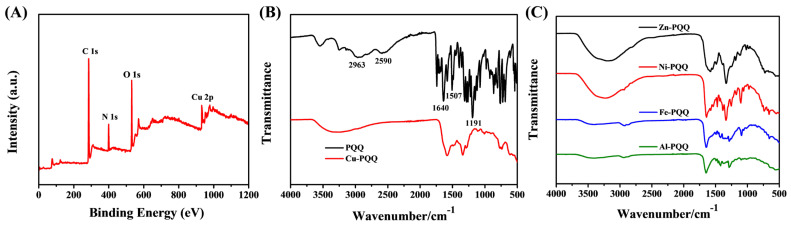
XPS survey (**A**) and FT-IR spectrum (**B**) of Cu-PQQ nanoparticles. (**C**) FT-IR spectra of Zn-PQQ, Ni-PQQ, Fe-PQQ, and Al-PQQ.

X-ray photoelectron spectroscopy (XPS) is a surface-sensitive quantitative spectroscopic technique that can identify the elements existing in a material. To further confirm the elemental compositions, Cu-PQQ nanoparticles were characterized by the XPS spectra. The XPS survey confirmed the presence of copper, carbon, nitrogen, and oxygen elements in the Cu-PQQ hybrids (Figure 2A). High-resolution spectra of Cu 2p and O 1s of the Cu-PQQ nanoparticles are shown in Supplementary Material. The Cu 2p of Cu-PQQ is decomposed into two peaks of 951.4 eV and 931.9 eV matched with Cu 2p_1/2_ and Cu 2p_3/2_, respectively (Figure S1A). The deconvoluted peaks at 934.1, 943.4, and 953.9 eV are assigned to the satellite peaks. This result suggested that the Cu element in the oxo-node of Cu-PQQ is mainly in the Cu(II) state. The O 1s spectrum for Cu-PQQ exhibits two peaks corresponding to 530.7 eV (C=O) and 531.9 eV (C–OH) (Figure S1B). In addition, the elemental compositions of Zn-PQQ, Ni-PQQ, Fe-PQQ, and Al-PQQ were also confirmed by their XPS spectra, as shown in Supplementary Material (Figure S2). Infrared spectroscopy is one of the fundamental techniques to confirm the existence of functional groups. The coordination of PQQ and metal ions was also confirmed by FT-IR spectra. As shown in Figure 2B,C, after coordination with metal ions, the relative intensity of the PQQ band at 2500~3000 cm^−1^, attributed to the stretching vibration of O–H and H bonds, decreased. The results suggested that metal ions were complexed with the carboxyl groups in PQQ molecules.

### 3.2. Stability of Cu-PQQ

**Figure 3:**
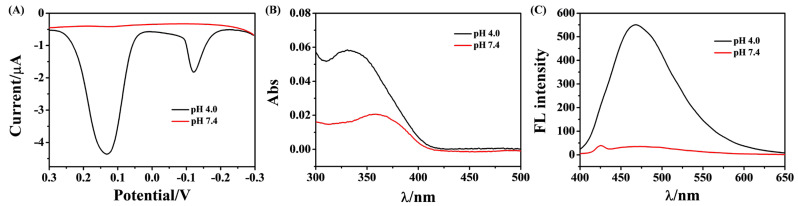
DPV responses (**A**), UV-vis spectra (**B**), and fluorescence spectra (**C**) of Cu-PQQ nanoparticles in pH 4.0 (black curve) and 7.4 (red curve) buffer solutions.

The metal coordination interaction is pH-dependent. Therefore, we investigated the stability of the Cu-PQQ nanoparticles in both acidic and neutral buffer solutions. The electrochemical, UV-vis, and fluorescent signals of the nanoparticles are shown in Figure 3. No or poor signals were observed in the neutral buffer solution. Two redox peaks were found in the pH 4.0 acetic acid buffer solution (Figure 3A). The oxidation peaks with potentials of 0.13 V and –0.12 V can be attributed to PQQ and Cu^2+^, respectively, which is consistent with those of pure PQQ and Cu^2+^ (Figure S3). After the dissolution of Cu-PQQ nanoparticles in an acid solution, the absorption and fluorescent intensity resulting from PQQ increased (Figure 3B,C) [26]. The Cu-PQQ nanoparticles showed a negligible fluorescent signal at pH 7.4, which is understandable since the fluorescence of PQQ can be quenched by the coordinated Cu^2+^ in the metal–organic assemblies through the ligand–metal charge transfer effect (Figure S4). We also investigated the effect of pH value on the acid-triggered release of PQQ and its peak current and fluorescence intensity (Figure S5). It was found that Cu-PQQ nanoparticles were very stable at pH values over 4.5, but they could be decomposed under acidic conditions. The result is understandable since the protonation of nitrogen and carboxyl groups can weaken the metal coordination interactions due to the competitive bonding of H^+^ with metal ions to coordinate with ligands [27]. As the pH influenced both the hydrolysis process of Cu-PQQ and the fluorescent emission of signal molecule PQQ, a pH 4.0 acetic acid buffer solution was selected for the following experiments. In addition, the molar ratio of Cu^2+^ and PQQ in the nanoparticles was calculated to be about 2.2:1 by determining the amounts of released Cu^2+^ and PQQ. The well-defined redox peak and excellent UV-vis and fluorescent signals of PQQ indicate that the Cu-PQQ nanoparticles can be used as signal labels for the development of triple-readout immunoassays based on the release of signaling molecules.

### 3.3. Characterization of the Interaction Between Cu-PQQ Nanoparticles and His_6_ Tags

**Figure 4:**
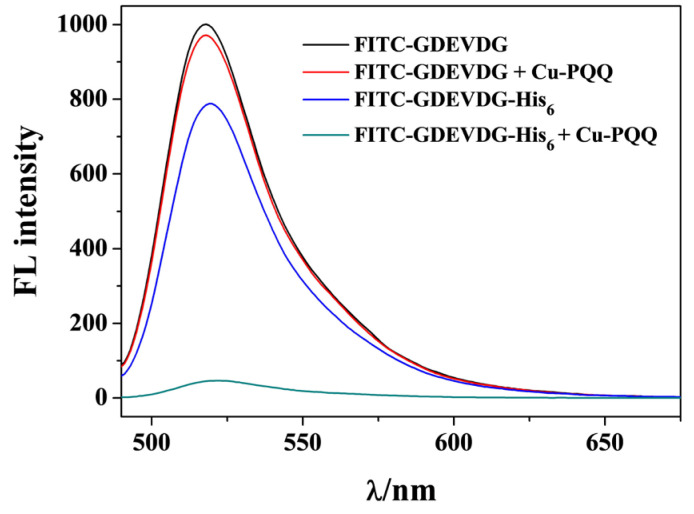
Fluorescence spectra of 1 μM FITC-GDEVDG and FITC-GDEVDG-His_6_ in the absence and presence of 10 μg/mL Cu-PQQ nanoparticles.

The site-specific and oriented immobilization of bioreceptors or recognition elements on a solid surface can favor intermolecular interactions. It has been demonstrated that Lewis bases, such as the imidazole group in the histidine (His) amino acid residue, can attach to the surface of metal–organic hybrids via coordination interactions with the unsaturated metal sites [28]. For the convenience of separation and purification, most of the expressed proteins in commercial kits are prepared using recombinant techniques, which endows the proteins with additional labels, such as a His_6_ tag. The non-covalent and strong interactions between metal complexes and His_6_ tags can facilitate the site-specific immobilization or labeling of proteins, allowing for the development of various optical and electrochemical biosensors [28]. For instance, metal complex-modified cellulose membranes and magnetic nanoparticles or beads have been applied to concentrate histidine-rich proteins, and metal complex-labeled His_6_-tagged proteins can promote the generation of detectable signals in diagnostics due to their unique optical, catalytic, electrochemical, and magnetic properties. Wuttke’s group suggested that His_6_-tagged proteins could be anchored on metal–organic framework nanoparticles via metal coordination interactions [25], which facilitated the modification of nanomaterials with biorecognition elements and promoted the development of various biosensors. Herein, we suggest that the recombinant recognition element rSA can be immobilized onto the surfaces of Cu-PQQ nanoparticles through metal coordination interactions. To demonstrate the successful attachment of rSA onto the surface of Cu-PQQ, the ζ-potential change was monitored by a Zetasizer. It was found that the ζ-potential of Cu-PQQ changed from –19.2 to –23.7 mV after the modification of rSA. In addition, to prove that His_6_-tagged proteins can be attached on the Cu-PQQ surface, dye-labeled peptides with and without a His_6_ tag were used as the fluorescence probes to monitor the metal coordination interactions. It was found that the fluorescence of fluorescein isothiocyanate (FITC)-labeled His_6_-tagged peptide named FITC-GDEVDG-His_6_ could be quenched by Cu-PQQ, but there was no significant change for that of non-His_6_-tagged peptide (FITC-GDEVDG) (Figure 4). The result suggested that His_6_-tagged biomolecules can be anchored on the Cu-PQQ surface through the metal coordination interactions, contributing to the design of biosensors.

### 3.4. Principle of Triple-Readout Immunoassays

**Scheme 1:**
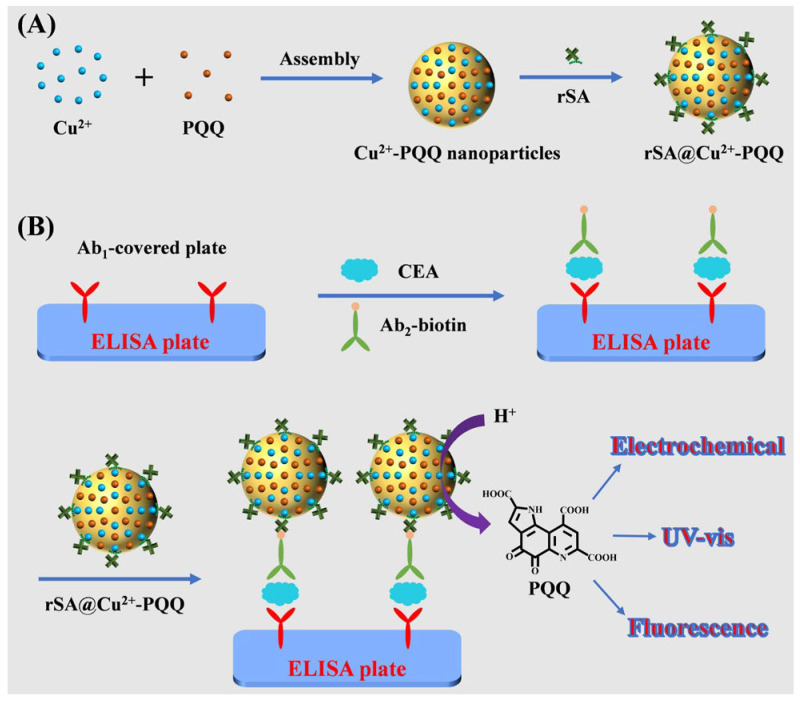
Schematic representation of Cu^2+^-coordinated self-assembly of PQQ (**A**) for triple-readout immunoassays of CEA (**B**).

Sandwich immunoassays exhibit high sensitivity, selectivity, and precision due to their low background signal and the use of a couple of matching antibodies. In this work, triple-readout immunoassays were designed in the sandwich detection format, with rSA@Cu-PQQ as the signal label to recognize biotinylated antibody. CEA is a non-specific serum biomarker that is highly expressed in many cancers (e.g., colorectal, medullary thyroid, breast, and mucinous ovarian). The level of CEA is crucial for the diagnosis and postoperative therapy of cancers. Therefore, CEA was used as a model analyte. Specifically, the nanolabels were prepared via the Cu^2+^-coordinated assembly of PQQ molecules (Scheme 1A). rSA was attached onto the surface of Cu-PQQ through the coordination interaction between the His_6_ tag in rSA and the unsaturated metal site on Cu-PQQ, serving as the linker of Ab_2_–biotin and Cu-PQQ via the classical avidin–biotin coupling system. The binding of CEA to the Ab_1_-covered microplate allowed for the capture of Ab_2_–biotin and rSA@Cu-PQQ. In an acidic condition, the captured Cu-PQQ labels will release a large number of PQQ molecules with electrochemical, UV-vis, and fluorescent signals, thus achieving signal-amplified, turn-on, and triple-readout immunoassays of CEA (Scheme 1B). The quantitative detection can be realized by directly converting the target concentrations into readable multiple signals in the same reaction system.

### 3.5. Feasibility of Triple-Readout Immunoassays

**Figure 5:**
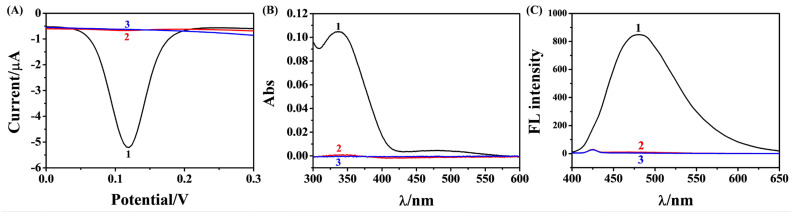
DPV responses (**A**), UV-vis spectra (**B**), and fluorescence spectra (**C**) obtained when the Ab_1_-covered microplates were treated by CEA/Ab_2_-biotin/rSA@Cu-PQQ (curve 1) or Ab_2_-biotin/rSA@Cu-PQQ (curve 2) and then incubated with pH 4.0 acetic acid buffer. Curve 3 corresponds to that of CEA/Ab_2_-biotin/rSA@Cu-PQQ after incubation with pH 7.4 phosphate buffer.

The feasibility of triple-readout immunoassays of CEA was investigated by incubating the Ab_1_-covered microplates with and without the target, followed by the capture of Ab_2_–biotin and rSA@Cu-PQQ and the addition of an acetic acid buffer solution. As shown in Figure 5, when the microplates were successively treated with CEA, Ab_2_–biotin, and CEA@Cu-PQQ and then incubated with the acetate buffer solution, well-defined electrochemical (Figure 5A), UV-vis (Figure 5B), and fluorescent (Figure 5C) signals from PQQ were observed (curve 1). However, no significant signals were obtained when the microplates were incubated with Ab_2_–biotin and rSA@Cu-PQQ without the capture of CEA (curve 2), indicating that both Ab_2_–biotin and rSA@Cu-PQQ showed poor, non-specific adsorption on the microplate surface. We also found that the peak current and signal intensity were dependent on the levels of Ab_2_–biotin and rSA@Cu-PQQ (Figure S6). This further demonstrated that the attachment of rSA@Cu-PQQ was dependent on the capture of CEA and Ab_2_–biotin. When the microplates covered with CEA, Ab_2_–biotin, and rSA@Cu-PQQ were treated with neutral buffer solutions, no significant signals were observed (curve 3). These results suggested that the Cu-PQQ nanoparticles were stable in a neutral but not in an acidic aqueous solution. Therefore, the Cu-PQQ nanoparticles can be used as labels for triple-readout immunoassays of CEA in a sandwich format.

### 3.6. Sensitivity for CEA Detection

**Figure 6:**
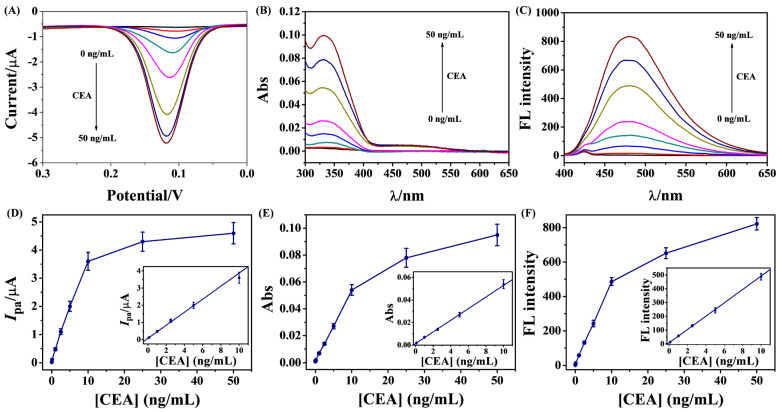
DPV responses (**A**), UV-vis spectra (**B**), and fluorescence spectra (**C**) for triple-readout immunoassays of CEA at different concentrations (0, 0.1, 1, 2.5, 5, 10, 25, and 50 ng/mL). Dependence of peak current (**D**), absorbance intensity (**E**), and fluorescence intensity (**F**) on CEA concentration. The inserts show the linear portions of the fitting curves.

The sensitivity of this method was evaluated by determining different concentrations of CEA. As shown in Figure 6A–C, the peak current and absorbance/fluorescence intensity were intensified as the CEA concentration increased. The linear equations in the concentration range of 0.1–10 ng/mL can be expressed as *I*_pa_ = 0.08 + 0.38 [CEA] (ng/mL) (DPV) (R^2^ = 0.996), Abs = 0.001 + 0.005 [CEA] (ng/mL) (UV-vis) (R^2^ = 0.999), and FL= 8 + 48 [CEA] (ng/mL) (fluorescence) (R^2^ = 0.999) (Figure 6D–F). The detection limits of the electrochemical, UV-vis, and fluorescent immunoassays were estimated to be 0.01, 0.1, and 0.1 ng/mL by determining the minimum target concentration that can be apparently distinguished from that of the background control. Among the three immunoassays, the detection limit of the electrochemical method was the lowest, which could be attributed to the well-defined electrochemical signal of PQQ molecule and the high sensitivity of electrochemical technique. Actually, we found that DPV method exhibited higher sensitivity for the determination of PQQ than UV-vis and fluorescence spectra. It is noticed that the UV-vis and fluorescent immunoassays exhibited similar detection limits, which is understandable since the two spectrographic techniques showed similar sensitivity for PQQ detection. The sensitivity is comparable to or even higher than that of previously reported immunoassays based on the release of redox metal ions or small dye molecules (Table 1). The relative standard deviations (RSDs) of the methods obtained in three repeated experiments were all lower than 10%, indicating acceptable reproducibility of the triple-readout immunoassays. All of the results suggested that the proposed method provided the triple-readout immunoassays with good reliability and sensitivity for target detection. In addition, copper is an indispensable trace element that serves as an intrinsic constituent of numerous natural enzymes, PQQ is an important redox-active molecule that can promote the oxidation of ascorbic acid, thiols, and other compounds via redox cycling or catalytic reactions [29,30,31,32,33], and nanozymes show distinct advantages over natural enzymes (e.g., cost-effectiveness, enhanced stability, and adjustable performance). These advantageous properties have inspired researchers to exploit different copper-based nanomaterials for catalytic applications [34]. In the presented work, the triple-readout immunoassays were conducted by directly monitoring the redox current of PQQ and its UV-vis as well as fluorescent signals. We believe that the sensitivity of immunoassays with Cu-PQQ nanoparticles as the signal labels could be further improved based on the dual catalytic sites of Cu^2+^ and PQQ.

### 3.7. Selectivity, Stability, and Recovery

**Figure 7:**
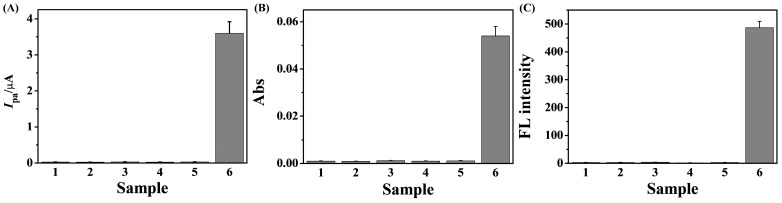
Selectivity of the DPV (**A**), UV-vis (**B**), and fluorescence (**C**) methods for the determination of different proteins (bar 1~6: blank, HSA, AFP, thrombin, PSA, and CEA). The concentration of CEA was 10 ng/mL, and that of other proteins was 100 ng/mL.

Selectivity and stability are two crucial factors for the practical application of immunoassays. A series of interfering proteins was introduced to evaluate the selectivity of the method. As shown in Figure 7, the signal intensities of the interfering proteins, such as human serum albumin (HSA), α-fetoprotein (AFP), thrombin, and prostate-specific antigen (PSA), at concentrations at least 10-fold higher than that of CEA, were close to those of the blank controls (bars 1~5). The target protein of CEA caused a significant increase in the peak current and absorbance/fluorescence intensity (bar 6). This is indicative of the excellent selectivity of the triple-readout immunoassays. In addition, the current value and absorbance intensity retained over 93% of the initial values when Cu-PQQ nanoparticles were stored at 4 °C for at least ten weeks, indicating the excellent stability of the signal labels. To further assess the accuracy and reliability of this method for real sample analysis, recovery experiments were conducted by spiking three concentrations of CEA standard samples into fetal bovine serum. As shown in Table 2, the recovery ranged from 97% to 120%, which is indicative of the minimized matrix effect of biological matrices on the triple-readout immunoassays. The proposed triple-mode biosensors show high throughput, provide multilevel detection data and reliable results, and obtain a greater amount of available information. However, they need three different devices to collect the detection signals, increasing the difficulty of their applications in clinical trials, taking into account analysis time, detection cost, and operational steps. We believe that portable devices with on-site detection capabilities will meet the needs of point-of-care test detection by directly converting the target concentrations into readable multiple signals in the same reaction system.

## 4. Conclusions

The multiple-signal detection mode has the advantages of a wide linear range, ease of self-verification, and improved detection diversity and precision. However, few studies for triple-mode immunoassays have been reported due to the lack of multifunctional sensing materials. In this work, a triple-readout immunoassay platform was developed with metal–organic hybrids as the signal labels based on the triple-signal property of PQQ. The coordination-driven self-assembly between Cu^2+^ and PQQ is simple, environmentally friendly, and time-saving. The preparation and modification procedures of Cu^2+^-PQQ nanoparticles are more convenient than those of other nanomaterials. The signaling molecules were released from the nanolabels to produce electrochemical, UV-vis, and fluorescent signals when adjusting the solution pH, thereby achieving signal-amplified sandwich immunoassays with CEA as a model analyte. The electrochemical method was more sensitive than the other strategies explored in this study. The simple and fast release of PQQ endowed this method with high sensitivity, closely approaching that of commercial ELISA kits. This indicates that our signal amplification strategy could, in principle, compete with ELISAs.

Although different assay modes can compensate for each other’s limitations, multiple-mode detection strategies still have significant room for improvement to meet the demands of practical sensing applications. For instance, it is essential to develop multimodal portable devices for efficient multi-signal detection and explore advanced nanomaterials with multifunctionality, uniform morphology, and low toxicity to enhance the detection accuracy and environmental friendliness of biosensors. Our group is dedicated to develop highly stable, low-cost, and environmentally friendly multimodal signal labels based on the self-assembly of small signaling molecules. In addition, we will exploit the catalytic properties of Cu-PQQ and develop novel biosensors based on the dual-catalytic sites of both Cu^2+^ and PQQ.

## Figures and Tables

**Table 1:** Analytical performances of different immunoassay methods achieved by determining the released signaling elements, including metal ions and small organic molecules.

Method	Signal Label	Releasing Agent	Target	Linear Range (ng/mL)	Detection Limit (ng/mL)	Ref.
SWV	Cd-based QDs	HNO_3_	PSA	0–20	0.12	[35]
SWV	Cd-based QDs	HCl	PSA	0.5–80	0.2	[36]
SWV	Cd-based QDs	HCl	OP-AChE	0.3–3 × 10^2^	0.15	[37]
ASV	Pb/Cd-based QDs	HCl	Casein, IgG	0–5 × 10^3^, 0–2 × 10^3^	40, 20	[38]
DPV	FcFNPs	Methanol	PSA	10^−3^–7.5 × 10^−2^	10^−3^	[39]
UV-vis	TP@MSN	NaOH	PSA	5 × 10^−4^–8	3.6 × 10^−4^	[40]
UV-vis	TP@MPDA	NaOH	AFP	10^−2^–1	2.3 × 10^−3^	[41]
UV-vis	PP@cC_3_N_4_	NaOH	CEA	0.5–10^−2^	3.4 × 10^−4^	[42]
UV-vis	PQQ@MSN	Phosphate	PSA	5 × 10^−3^–0.5	10^−3^	[43]
UV-vis	TMB NPs	Ethanol	IL-6	1 × 10^−3^–1	6.6 × 10^−4^	[44]
fluorescence	FL@liposome	Not reported	IgG	10^−10^–10^−7^	2 × 10^−3^	[45]
fluorescence	FL@AuNF	NaOH	AFP	10^−5^–10^−2^	2.9 × 10^−8^	[46]
fluorescence	SRB@liposome	OG	CT	10^−10^–10^−8^	6 × 10^−11^	[47]
fluorescence	C153@PSP	Ethanol	CRP	12–45	4.9	[48]
fluorescence	FDA NCs	DMSO	IgG	0–2	5.7 × 10^−2^	[49]
fluorescence	TCPP NPs	NaOH	IgG	1.5–1.5 × 10^2^	0.31	[50]
fluorescence	bio-PyFNPs	Methanol	AFP	10^−3^–2.5	5 × 10^−4^	[51]
DPV, UV-vis, fluorescence	Cu-PQQ	Acetic acid	CEA	0.1–10	0.01, 0.1, 0.1	This work

Abbreviations: SWV, square wave voltammetry. QDs, quantum dots. PSA, prostate-specific antigen. OP-AChE, organophosphorylated acetylcholinesterase. ASV, anodic stripping voltammetry. DPV, differential pulse voltammetry. FcFNPs, ferrocenoyl phenylalanine nanoparticles. TP, thymolphthalein. MSN, mesoporous silica nanoparticles. MPDA, metal–polydopamine framework. AFP, alpha-fetoprotein. PP, phenolphthalein. CEA, carcinoembryonic antigen. PQQ, pyrroloquinoline quinone. TMB NPs, 3,3′,5,5′-tetramethylbenzidine nanoparticles. IL-6, Interleukin-6. FL, fluorescein. AuNF, gold nanoflower. SRB, sulforhodamine B. OG, n-octyl β-D-glucopyranoside. CT, cholera toxin. PSP@C153, coumarin153-stained polystyrene particles. CRP, C-reactive protein. FDA NCs, fluorescein diacetate nanocrystals. TCPP NPs, tetra(4-carboxyphenyl)porphyrin nanoparticles. bio-PyFNPs, biotinylated pyrene-phenylalanine nanoparticles.

**Table 2:** Results for the triple-model immunoassays of CEA spiked in fetal bovine serum.

Added (ng/mL)		Found (ng/mL)	
	DPV	UV-Vis	Fluorescence
0.10	0.11 ± 0.01	0.12 ± 0.01	0.12 ± 0.01
1.00	1.04 ± 0.10	1.06 ± 0.13	1.08 ± 0.12
5.00	4.86 ± 0.38	4.92 ± 0.41	5.02 ± 0.39

## Data Availability

Data are contained within the article.

## Supplementary Materials

The following supporting information can be downloaded at: https://www.mdpi.com/article/10.3390/bios15110710/s1. Reagents and Apparatus. Figure S1. High-resolution spectra of Cu 2p and O 1s for the synthesized Cu-PQQ nanoparticles. Figure S2. XPS survey of Zn-PQQ, Ni-PQQ, Fe-PQQ, and Al-PQQ. Figure S3. DPV responses of pure PQQ and Cu^2+^. Figure S4. Fluorescence spectra of PQQ and PQQ/Cu^2+^ mixture. Figure S5. Effect of pH value on the peak current and fluorescence intensity of PQQ and Cu-PQQ. Figure S6. Dependence of fluorescence intensity on Ab2-biotin concentration for the assays of CEA.
