# Supplementary material for: Triple-Model Immunoassays with the Self-Assemblies of Three-in-One Small Molecules as Signaling Labels
Source: Biosensors (Basel). 2025 Oct 24;15(11):710. doi: 10.3390/bios15110710 (PMC12650134; doi:10.3390/bios15110710)
Supplement: Supplementary file 1 [file biosensors-15-00710-s001.zip › biosensors-3905518-supplementary.pdf]

Triple-Model Immunoassays with the Self-Assemblies of Three-in-One Small Molecules as Signaling Labels

Zhaojiang Yu, Wenqi Yuan, Mingyi Qiao and Lin Liu*

College of Chemistry and Chemical Engineering, Anyang Normal University, Anyang, Henan 455000, China

*Correspondence: liulin@aynu.edu.cn

1. Reagents and Apparatus

Human serum albumin (HSA), α-fetoprotein (AFP), and thrombin were ordered from Sigma-Aldrich Company (Shanghai, China). Prostate-specific antigen (PSA), carcinoembryonic antigen (CEA), capture antibody (Ab_1)-covered 96-well microplates, and biotinylated detection antibody (Ab_2–biotin) were provided by Linc-Bio Science Co. Ltd (Shanghai, China). Recombinant streptavidin (rSA) was produced by ProSpec-Tany TechnoGene Ltd. (Ness-Ziona, Israel). Peptides were synthesized by China Peptides Co., Ltd (Shanghai, China). PQQ, polyvinyl pyrrolidone (PVP), and other reagents were obtained from Aladdin Chemistry Co., Ltd. (Shanghai, China). Fetal bovine serum was ordered from Gibco Company (USA). All chemical reagents were of analytical grade and used without any purification. Millipore ultrapure water was used for the preparation of all aqueous solutions.

The morphologies of metal–PQQ hybrids were characterized using a scanning electron microscope (SEM, JSM-IT800, Japan) and an FEI Tecnai G2 T20 transmission electron microscope (TEM, Hillsboro, OR, USA). Element analysis pictures were collected with an energy-dispersive X-ray spectroscopy device (EDS, Bruker, Quantax) equipped on the SEM. ζ-Potential was measured using a Nano ZS90 Zetasizer (Malver Instruments Ltd., U.K.). X-ray photoelectron spectroscopy (XPS) was performed with a Thermo ESCALAB 250Xi instrument. The Fourier transform infrared (FT-IR) spectra were collected using a NEXUS-470 spectrometer. Fluorescence data were collected using a Hitachi F-4600 fluorescence spectrometer (Hitachi High-Tech, Japan) with 5 nm excitation and emission slit widths. UV–vis spectra were collected using a Cary 60 spectrophotometer (Santa Clara, USA). Electrochemical measurements were conducted using a CHI660E electrochemical workstation (CH Instruments Inc., China) with a glass carbon electrode (3 mm diameter) as the working electrode. A platinum wire and a Ag/AgCl electrode were used as the auxiliary and reference electrodes, respectively.

2. Results

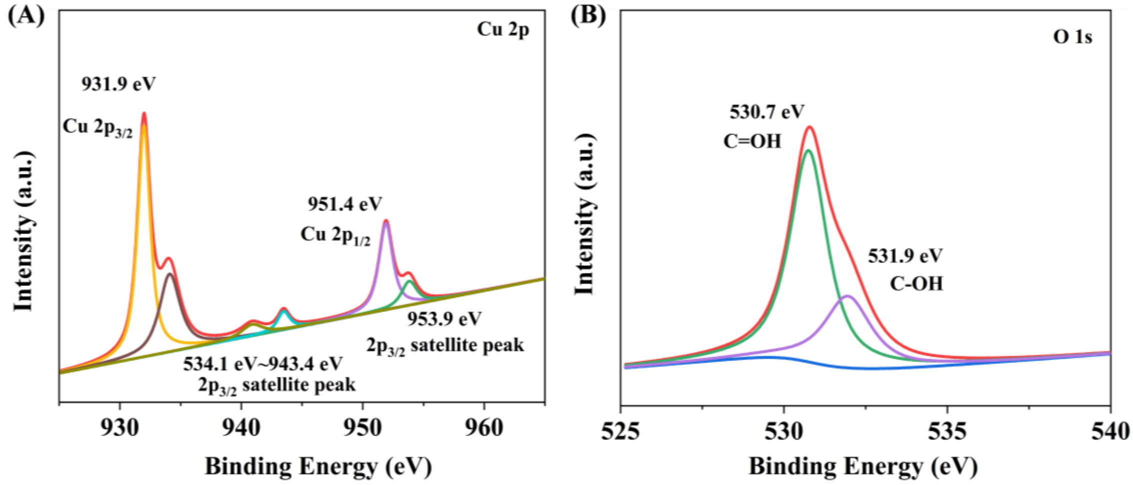

Figure S1. High-resolution spectra of (**A**) Cu 2p and (**B**) O 1s for the synthesized Cu-PQQ nanoparticles.

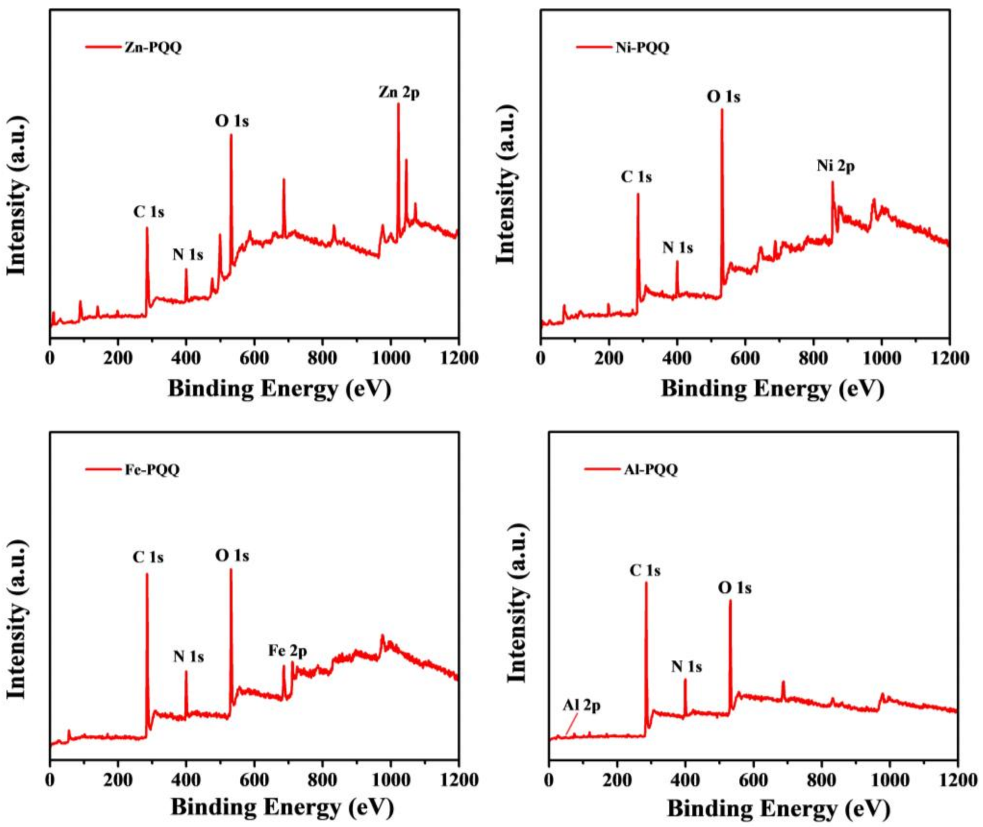

Figure S2. XPS survey of Zn-PQQ, Ni-PQQ, Fe-PQQ, and Al-PQQ.

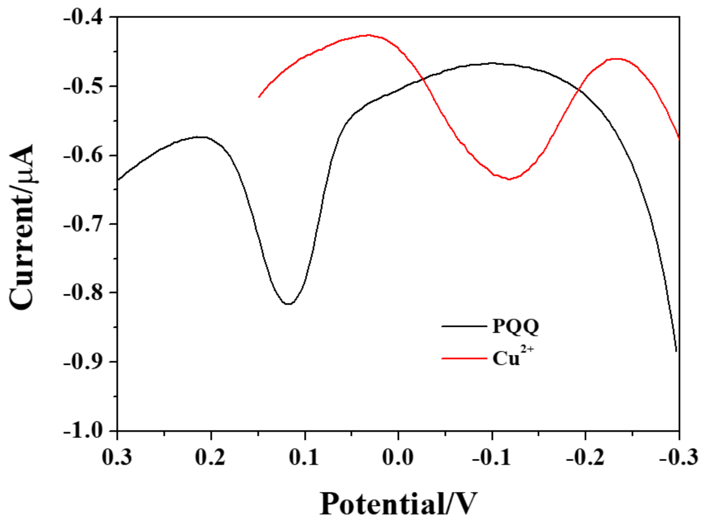

Figure S3. DPV responses of 0.1 μM pure PQQ (black curve) and Cu^{2+} (red curve) in pH 4.0 acetic acid buffer solution.

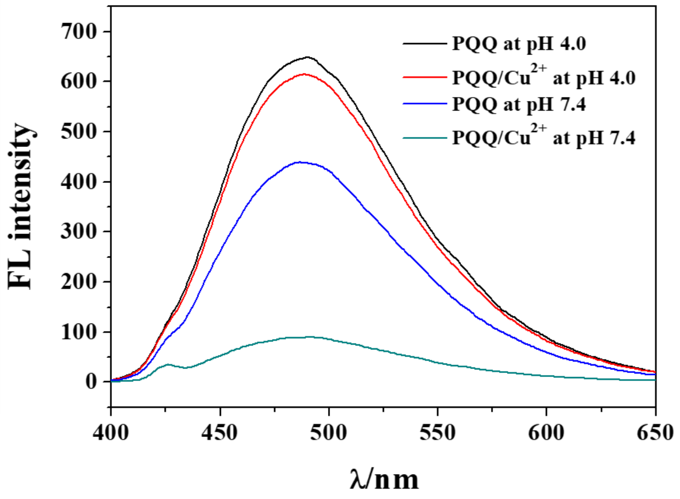

Figure S4. Fluorescence spectra of 5 μM pure PQQ and PQQ/Cu^{2+} mixture in pH 4.0 acetic acid buffer and pH 7.4 phosphate buffer.

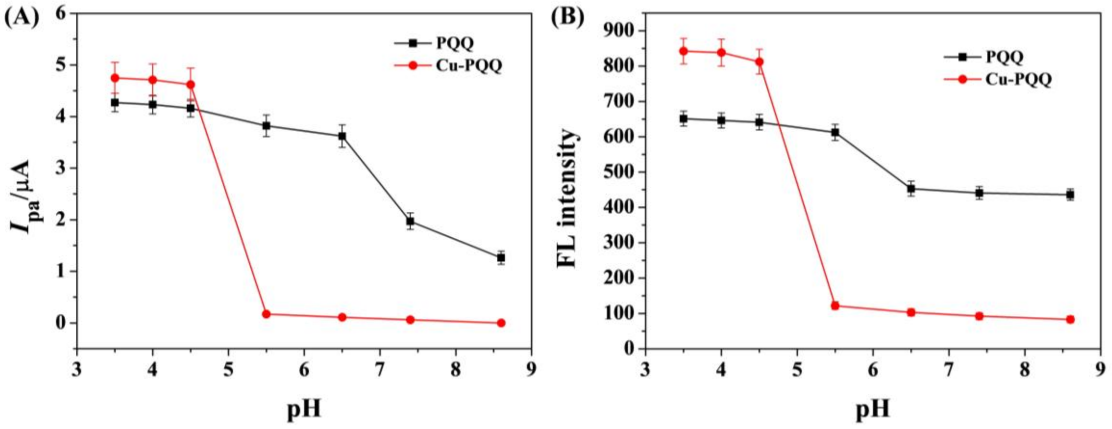

Figure S5. Effect of pH value on the peak current (**A**) and fluorescence intensity (**B**) of 5 μM PQQ and 3.3 μg/mL Cu-PQQ.

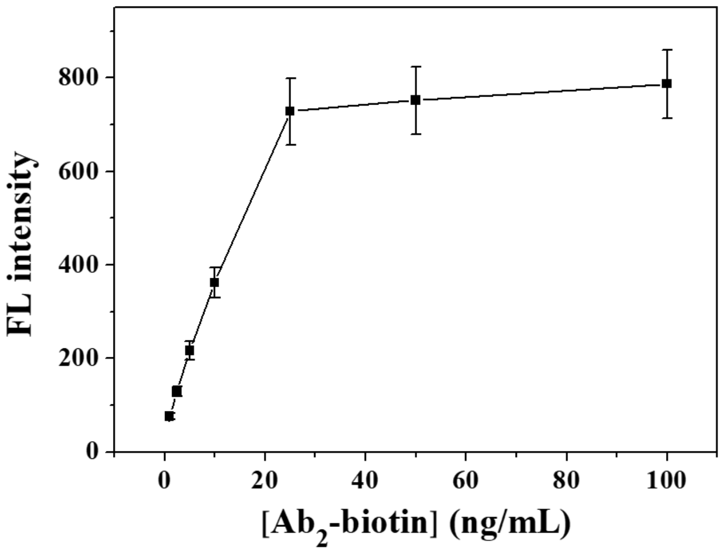

Figure S6. Dependence of fluorescence intensity on Ab_2-biotin concentration for the assays of 50 ng/mL CEA.
